# The Systemic Lupus Erythematosus *IRF5* Risk Haplotype Is Associated with Systemic Sclerosis

**DOI:** 10.1371/journal.pone.0054419

**Published:** 2013-01-23

**Authors:** F. David Carmona, Jose-Ezequiel Martin, Lorenzo Beretta, Carmen P. Simeón, Patricia E. Carreira, José Luis Callejas, Mónica Fernández-Castro, Luis Sáez-Comet, Emma Beltrán, María Teresa Camps, María Victoria Egurbide, the Spanish Scleroderma Group, Paolo Airó, Raffaella Scorza, Claudio Lunardi, Nicolas Hunzelmann, Gabriela Riemekasten, Torsten Witte, Alexander Kreuter, Jörg H. W. Distler, Rajan Madhok, Paul Shiels, Jacob M. van Laar, Carmen Fonseca, Christopher Denton, Ariane Herrick, Jane Worthington, Annemie J. Schuerwegh, Madelon C. Vonk, Alexandre E. Voskuyl, Timothy R. D. J. Radstake, Javier Martín

**Affiliations:** 1 Instituto de Parasitología y Biomedicina López-Neyra, CSIC, Granada, Spain; 2 Referral Center for Systemic Autoimmune Diseases, Fondazione IRCCS Ca' Granda Ospedale Maggiore Policlinico di Milano, University of Milan, Milan, Italy; 3 Department of Internal Medicine, Hospital Vall d’Hebron, Barcelona, Spain; 4 Department of Rheumatology, Hospital 12 de Octubre, Madrid, Spain; 5 Department of Internal Medicine, Hospital Clínico San Cecilio, Granada, Spain; 6 Department of Rheumatology, Hospital Puerta de Hierro Majadahonda, Madrid, Spain; 7 Department of Internal Medicine, Hospital Universitario Miguel Servet, Zaragoza, Spain; 8 Department of Rheumatology, Hospital General Universitario de Valencia, Valencia, Spain; 9 Department of Internal Medicine, Hospital Carlos Haya, Málaga, Spain; 10 Department of Internal Medicine, Hospital de Cruces, Barakaldo, Spain; 11 Servizio di Reumatologia ed Immunologia Clinica Spedali Civili, Brescia, Italy; 12 Department of Medicine, Università degli Studi di Verona, Verona, Italy; 13 Department of Dermatology, University of Cologne, Cologne, Germany; 14 Department of Rheumatology and Clinical Immunology, Charité University Hospital, Berlin, Germany; 15 Hannover Medical School, Hannover, Germany; 16 Ruhr University of Bochum, Bochum, Germany; 17 Department of Internal Medicine 3, Institute for Clinical Immunology, University of Erlangen-Nuremberg, Erlangen, Germany; 18 Centre for Rheumatic Diseases, Glasgow Royal Infirmary, University of Glasgow, Glasgow, United Kingdom; 19 Institute of Cellular Medicine, Newcastle University, Newcastle, United Kingdom; 20 Centre for Rheumatology, Royal Free and University College Medical School, London, United Kingdom; 21 Arthritis Research UK Epidemiology Unit, University of Manchester, Manchester Academic Health Science Centre, Manchester, United Kingdom; 22 Department of Rheumatology, Leiden University Medical Center, Leiden, The Netherlands; 23 Department of Rheumatology, Radboud University Nijmegen Medical Center, Nijmegen, The Netherlands; 24 Department of Rheumatology, VU University Medical Center, Amsterdam, The Netherlands; 25 Department of Rheumatology and Clinical Immunology, University Medical Center Utrecht, Utrecht, The Netherlands; Keio University School of Medicine, Japan

## Abstract

Systemic sclerosis (SSc) is a fibrotic autoimmune disease in which the genetic component plays an important role. One of the strongest SSc association signals outside the human leukocyte antigen (HLA) region corresponds to interferon (IFN) regulatory factor 5 (*IRF5*), a major regulator of the type I IFN pathway. In this study we aimed to evaluate whether three different haplotypic blocks within this *locus*, which have been shown to alter the protein function influencing systemic lupus erythematosus (SLE) susceptibility, are involved in SSc susceptibility and clinical phenotypes. For that purpose, we genotyped one representative single-nucleotide polymorphism (SNP) of each block (rs10488631, rs2004640, and rs4728142) in a total of 3,361 SSc patients and 4,012 unaffected controls of Caucasian origin from Spain, Germany, The Netherlands, Italy and United Kingdom. A meta-analysis of the allele frequencies was performed to analyse the overall effect of these *IRF5* genetic variants on SSc. Allelic combination and dependency tests were also carried out. The three SNPs showed strong associations with the global disease (rs4728142: *P*  = 1.34×10^−8^, OR  = 1.22, CI 95%  = 1.14–1.30; rs2004640: *P*  = 4.60×10^−7^, OR  = 0.84, CI 95%  = 0.78–0.90; rs10488631: *P*  = 7.53×10^−20^, OR  = 1.63, CI 95%  = 1.47–1.81). However, the association of rs2004640 with SSc was not independent of rs4728142 (conditioned *P*  = 0.598). The haplotype containing the risk alleles (rs4728142*A-rs2004640*T-rs10488631*C: *P*  = 9.04×10^−22^, OR  = 1.75, CI 95%  = 1.56–1.97) better explained the observed association (likelihood *P*-value  = 1.48×10^−4^), suggesting an additive effect of the three haplotypic blocks. No statistical significance was observed in the comparisons amongst SSc patients with and without the main clinical characteristics. Our data clearly indicate that the SLE risk haplotype also influences SSc predisposition, and that this association is not sub-phenotype-specific.

## Introduction

Systemic sclerosis (SSc) is a chronic multisystem connective tissue disorder characterized by fibrotic events, vascular damage and autoantibody production. Two main clinical subtypes have been defined based on the extent of skin involvement, limited cutaneous scleroderma (lcSSc) and diffuse cutaneous scleroderma (dcSSc) [1]. Recent candidate gene and genome-wide association studies (GWASs) clearly suggest that an important genetic component underlies this disease. In this regard, an increasing number of *loci* have been reported to be convincingly associated with the susceptibility and clinical manifestations of SSc in the last years. However, the causal functional mutations responsible for these associations have not been unambiguously identified yet in most cases [2].

Outside the HLA region, interferon (IFN) pathway genes, which encode cytokines with critical modulatory effects on innate and adaptive immunity, have been shown to represent a key component of the genetic network leading to autoimmune processes. Interestingly, a misregulated expression of type I IFN genes, also referred to as IFN signature, have been observed in peripheral white blood cells patient subsets of several autoimmune diseases [3], [4], [5], [6], thus suggesting that the IFN signaling plays a crucial role in autoimmunity. Indeed, multiple single-nucleotide polymorphisms (SNPs) of the IFN regulatory factor 5 gene (*IRF5*), a major regulator of the type I IFN induction, have been associated with different rheumatic disorders such as SSc, systemic lupus erythematosus (SLE), rheumatoid arthritis (RA), and Sjögren’s syndrome (SS) [7], [8], [9], [10]. The *IRF5* association with SLE was narrowed down to three different haplotype blocks that seem to have independent functional consequences, including 1) alteration of the protein stability, 2) creation of a donor splice site in intron 1 resulting in transcription of an alternative exon 1B, and 3) modification of the 3′UTR length which affects expression levels [11]. Subsequent studies in SSc patients suggested that genetic variation within *IRF5* correlate with SSc severity and survival [12], [13].

Based on the above, we decided to explore whether the functional haplotype blocks described by Graham *et al*. [11] were also susceptibility signals affecting SSc development and progression. For that purpose, we analysed the allele frequencies of three representative *IRF5* genetic variants that have been previously associated with SSc [8], [14] in five large Caucasian European cohorts and performed allelic combination and dependency tests.

## Patients and Methods

### Study Population

We recruited a total of 3,361 SSc patients and 4,012 unaffected controls of Caucasian origin from five different European countries, including an initial cohort from Spain and four replication cohorts from Germany, The Netherlands, Italy and United Kingdom. Case and control sets were matched by geographical origin and ethnicity. Written informed consent from all participants and approval from the local ethical committees of all centres involved in the study were obtained in accordance with the tenets of the Declaration of Helsinki. All SSc patients fulfilled the classification criteria by Leroy *et al*. [15]. The clinical features of the different SSc cohorts are shown in **Table 1**. Case sets were further subdivided based on their skin involvement into limited cutaneous scleroderma (lcSSc) and diffuse cutaneous scleroderma (dcSSc) subgroups [15], and by autoantibody status according to the presence/absence of anti-centromere antibodies (ACA) or anti-topoisomerase antibodies (ATA), which were detected using standard procedures. Pulmonary fibrosis (PF) was diagnosed by high resolution computed tomography (HRCT).

**Table 1:** Main clinical features of systemic sclerosis patients included in the study.

	N (%)				
Feature	Spain	Germany	Netherlands	Italy	UK
Female	1089 (89.14)	494 (85.83)	323 (81.83)	644 (91.50)	388 (83.33)
Male	133 (10.86)	81 (14.17)	72 (18.17)	60 (8.50)	77 (16.67)
lcSSc	843 (68.99)	338 (58.78)	271 (68.61)	515 (73.15)	336 (72.26)
dcSSc	379 (31.01)	237 (41.22)	124 (31.39)	189 (26.85)	129 (27.74)
ACA+	560 (45.83)	214 (37.22)	99 (25.06)	312 (44.32)	169 (36.34)
ACA-	617 (50.49)	341 (59.30)	291 (73.67)	385 (54.69)	285 (61.29)
ATA+	267 (21.85)	174 (30.26)	106 (26.84)	238 (33.81)	71 (15.27)
ATA-	881 (72.09)	376 (65.39)	284 (71.90)	461 (65.48)	382 (82.15)
PF+	294 (24.06)	175 (30.43)	144 (36.46)	205 (29.12)	111 (23.87)
PF-	830 (67.92)	327 (56.87)	158 (40.00)	400 (56.82)	189 (40.65)

Data are referred to the total analysed individuals.

ACA; anti-centromere antibodies; ATA, anti-topoisomerase antibodies; dcSSc, difusse cutaneous systemic sclerosis; lcSSc, limited cutaneous systemic sclerosis; PF, pulmonary fibrosis.

### SNPs Selection and Genotyping

Samples were genotyped for three *IRF5* tag SNPs, rs10488631, rs2004640, and rs4728142, representative of three different haplotype blocks (refers to as Groups 1–3, respectively) which have been reported to have functional roles in SLE patients [11]: Group 1 includes SNPs tagging a 30-bp in-frame INDEL variant of exon 6 that alters protein stability; Group 2 includes an exon 1B splice site variant; and Group 3 corresponds to genetic variants located in a conserved polyadenilation signal sequence that alters the length of the 3′UTR, thus affecting expression levels.

Genomic DNA was obtained from peripheral blood cells using standard procedures, and genotyping was performed using TaqMan® 5′ allele discrimination assays (IDs: C___2691242_10, C___9491614_10, and C___2691222_10), in a 7900 HT Fast Real-Time PCR System (Applied Biosystems, Foster City, California, USA).

### Statistical Analysis

The statistical power of the study was calculated with Power Calculator for Genetic Studies 2006 software (http://www.sph.umich.edu/csg/abecasis/CaTS/reference.html), which implements the methods described in Skol *et al*. [16].

PLINK v1.07 (http://pngu.mgh.harvard.edu/purcell/plink/) [17] was used to carried out all statistical analyses of allele frequencies. *P*-values were obtained by performing 2×2 contingency tables and χ^2^ test and/or Fisher’s exact test, when appropriate. Since the association between *IRF5* and SSc has been confirmed in several independent studies [2], we considered appropriate to set the significance threshold at *P  = *0.05. Odds ratios (OR) and 95% confidence intervals were calculated according to Woolf’s method. Breslow–Day (BD) test method was used to estimate the homogeneity amongst populations. Pooled analyses were performed by Mantel-Haenszel test under fixed effects, or DerSimonian-Laird if the BD test reached statistical significance.

Dependency of association between the studied genetic variants was determined by conditional logistic regression analysis as implemented in PLINK, and the allelic combinations were tested using PLINK, StatsDirect (V.2.6.6; StatsDirect, Altrincham, UK), and Haploview (V.4.2) [18].

To analyse whether allelic combinations would better explained the possible association than the genetic variants independently, we compared the goodness of fit of both models using PLINK. For that purpose, we calculated the deviance (defined as −2 × the log likelihood), which follows a χ^2^ distribution, to assess the significance of the improvement in fit. If statistically significant differences in the improvement of fit were observed when the haplotype effect was considered, we assumed that this model was more informative explaining the putative association.

## Results

The overall statistical power of the study, based on previous *IRF5* reports, to detect associations with OR  = 1.2 at 0.05 significance level was 100% for rs2004640 and rs4728142, and 93% for rs10488631 (**Table S1 in File S1**). Additionally, no significant departure from Hardy-Weinberg equilibrium was observed either in cases or controls in each analysed population (*P*  = 0.05).

### Allele Test

The results of the global analyses of the discovery cohort and the four independent replication populations separately are shown in **Table S2 in File S1**. Since the Breslow-Day test evidenced no heterogeneity of the ORs amongst the different cohorts (*P*  = 0.05), a combined meta-analysis was performed to test the overall effect of the *IRF5* genetic variants in the whole dataset (**Table 2**). The pooled analysis showed that the three SNPs were strongly associated with the global disease (rs4728142: *P*  = 1.34×10^−8^, OR  = 1.22, CI 95%  = 1.14–1.30; rs2004640: *P*  = 4.60×10^−7^, OR  = 0.84, CI 95%  = 0.78–0.90; rs10488631: *P*  = 7.53×10^−20^, OR  = 1.63, CI 95%  = 1.47–1.81). Highly significant *P*-values were also yielded when the different phenotype subgroups were compared against the control population (**Table S3 in File S1**). However, no statistical significance was observed in the comparisons amongst SSc patients accordingly with the presence/absence of the different clinical characteristics and autoantibody profile (**Table 2**), *i.e.* lcSSc vs. dcSSc (rs4728142: *P*  = 0.564; rs2004640: *P*  = 0.971; rs10488631: *P*  = 0.086), SSc ACA+ vs. SSc ACA- (rs4728142: *P*  = 0.359; rs2004640: *P*  = 0.357; rs10488631: *P*  = 0.449), SSc ATA+ vs. SSc ATA- (rs4728142: *P*  = 0.154; rs2004640: *P*  = 0.128; rs10488631: *P*  = 0.259), and SSc PF+ vs. SSc PF- (rs4728142: *P*  = 0.934; rs2004640: *P*  = 0.397; rs10488631: *P*  = 0.945), thus indicating the three *IRF5* polymorphisms are indeed associated with the global disease and there was no phenotype-specific association.

**Table 2:** Meta-analysis of *IRF5* genetic variants accordingly with the global disease and the presence or absence of the main clinical features.

				Genotype, N (%)				M-H allele test		
SNP	Position	1/2	Subgroup (N)	1/1	1/2	2/2	MAF (%)	*P*-value	OR [CI 95%]†	*P_B_*_D_ ¶
rs4728142	3′UTR	A/G	Controls (n = 3933)	787 (20.01)	1924 (48.92)	1222 (31.07)	44.47	**1.34E-08**	1.22 [1.14–1.30]	0.32
			SSc (n = 3128)	769 (24.58)	1549 (49.52)	810 (25.90)	49.34			
			lcSSc (n = 2142)	533 (24.88)	1059 (49.44)	550 (25.68)	49.60	0.564	0.97 [0.87–1.08]	0.61
			dcSSc (n = 986)	236 (23.94)	490 (49.70)	260 (26.37)	48.78			
			ACA- (n = 1781)	444 (24.93)	852 (47.84)	485 (27.23)	48.85	0.359	1.05 [0.95–1.16]	0.08
			ACA+ (n = 1268)	307 (24.21)	658 (51.89)	303 (23.90)	50.16			
			ATA- (n = 2222)	531 (23.90)	1110 (49.95)	581 (26.15)	48.87	0.154	1.09 [0.97–1.22]	0.28
			ATA+ (n = 793)	215 (27.11)	378 (47.67)	200 (25.22)	50.95			
			PF- (n = 1797)	427 (23.76)	929 (51.70)	441 (24.54)	49.61	0.934	1.00 [0.89–1.12]	0.97
			PF+ (n = 893)	237 (26.54)	409 (45.80)	247 (27.66)	49.44			
rs2004640	Exon 1	G/T	Controls (n = 3912)	897 (22.93)	1895 (48.44)	1120 (28.63)	47.15	**4.60E-07**	0.84 [0.78–0.90]	0.12
			SSc (n = 3122)	584 (18.71)	1511 (48.40)	1027 (32.90)	42.91			
			lcSSc (n = 2143)	406 (18.95)	1026 (47.88)	711 (33.18)	42.88	0.971	1.00 [0.90–1.12]	0.23
			dcSSc (n = 979)	178 (18.18)	485 (49.54)	316 (32.28)	42.95			
			ACA- (n = 1772)	354 (19.98)	833 (47.01)	585 (33.01)	43.48	0.357*	0.91 [0.74–1.12]	0.01
			ACA+ (n = 1272)	211 (16.59)	643 (50.55)	418 (32.86)	41.86			
			ATA- (n = 2216)	424 (19.13)	1076 (48.56)	716 (32.31)	43.41	0.128	0.91 [0.81–1.03]	0.43
			ATA+ (n = 793)	137 (17.28)	377 (47.54)	279 (35.18)	41.05			
			PF- (n = 1800)	327 (18.17)	899 (49.94)	574 (31.89)	43.14	0.397	0.95 [0.85–1.07]	0.84
			PF+ (n = 883)	163 (18.46)	417 (47.23)	303 (34.31)	42.07			
rs10488631	INDEL tagger	C/T	Controls (n = 3958)	41 (1.04)	625 (15.79)	3292 (83.17)	8.93	**7.53E-20**	1.63 [1.47–1.81]	0.11
			SSc (n = 3148)	70 (2.22)	749 (23.79)	2329 (73.98)	14.12			
			lcSSc (n = 2165)	46 (2.12)	494 (22.82)	1625 (75.06)	13.53	0.086	1.14 [0.98–1.33]	0.96
			dcSSc (n = 983)	24 (2.44)	255 (25.94)	704 (71.62)	15.41			
			ACA- (n = 1798)	48 (2.67)	425 (23.64)	1325 (73.69)	14.49	0.449	0.94 [0.81–1.10]	0.92
			ACA+ (n = 1273)	21 (1.65)	306 (24.04)	946 (74.31)	13.67			
			ATA- (n = 2236)	48 (2.15)	508 (22.72)	1680 (75.13)	13.51	0.259*	1.17 [0.89–1.54]	0.03
			ATA+ (n = 799)	21 (2.63)	212 (26.53)	566 (70.84)	15.89			
			PF- (n = 1818)	43 (2.37)	425 (23.38)	1350 (74.26)	14.05	0.945	0.99 [0.84–1.17]	0.94
			PF+ (n = 886)	19 (2.14)	213 (24.04)	654 (73.81)	14.16			

† Odds ratio for the minor allele.

¶ Breslow-Day *P*-value.

* DerSimonian–Laird random effects model *P*-value. SSc, systemic sclerosis; lcSSc, limited cutaneous SSc; dcSSc, diffuse cutaneous SSc; ACA, anti-centromere antibodies; ATA, anti-topoisomerase antibodies. M-H, Mantel-Haenszel test under fixed effect.

### Conditional Logistic Regression

We decided to perform pairwise conditioning analyses to test whether there could be any dependency amongst them (**Table 3**). The analysis showed that every SNP maintained its statistical significance after conditioning to the other two except for rs2004640, which was dependent of rs4728142. The moderate linkage disequilibrium between them (r2∼0.68) could explain this fact (**Table S4 in File S1**).

**Table 3:** Conditional logistic regression analysis for the *IRF5* polymorphisms considering the five populations as covariate.

SNP	*P*-value	*P*-value:add to rs10488631	rs10488631 *P*-value:add to SNP	*P*-value:add to rs2004640	rs2004640 *P*-value:add to SNP
rs4728142	**1.344E-08**	**2.57E-04**	**8.92E-16**	**2.24E-03**	0.598
rs2004640	**4.603E-07**	**0.020**	**1.72E-16**	NA	NA
rs10488631	**7.528E-20**	NA	NA	**1.72E-16**	**0.020**

LD, linkage disequilibrium; SNP, single-nucleotide polymorphism.

### Haplotype Analysis

We also analysed the allelic combinations of the *IRF5* genetic variants included in this study according to the global disease (**Table 4**). The most associated haplotype was that containing the risk alleles of the three SNPs (rs4728142*A-rs2004640*T-rs10488631*C: *P*  = 9.04×10^−22^, OR  = 1.75, CI 95%  = 1.56–1.97). The protective haplotype also showed a very significant *P*-value (rs4728142*G-rs2004640*G-rs10488631*T: *P*  = 2.48×10^−7^, OR  = 0.84, CI 95%  = 0.78–0.89).

**Table 4:** Pooled-analysis of different allelic combinations of the *IRF5* genomic region according to disease.

Alleliccombination	Freq.Controls	Freq.Cases	*P*-value	OR [95% CI]
GGT	0.4612	0.4181	**2.48E-07**	0.84 [0.78–0.89]
ATT	0.3593	0.3652	0.533	1.02 [0.95–1.10]
ATC	0.0731	0.1215	**9.04E-22**	1.75 [1.56–1.97]
GTT	0.0777	0.0652	**4.47E-03**	0.83 [0.72–0.94]
GTC	0.0155	0.0187	0.153	1.21 [0.93–1.57]

Order of the SNPs: rs4728142*A/G | rs2004640*G/T | rs10488631*C/T.

OR, odds ratio.

When comparing the haplotype model with the independent SNP model, we observed a statistically significant improvement of the goodness of fit compared to rs4728142 (likelihood *P*-value  = 1.23×10^−17^), rs2004640 (likelihood *P*-value  = 1.94×10^−19^), or rs10488631 (likelihood *P*-value  = 1.48×10^−4^) individually.

On the other hand, we also performed a sub-phenotype analysis of allelic combinations to test whether some haplotype could influence a specific clinical condition (**Table S5 in File S1**). This analysis only showed a residual *P*-value for a low frequency haplotype in the PF+/PF- comparison (rs4728142*G-rs2004640*T-rs10488631*T: *P*  = 0.041, OR  = 1.28, CI 95%  = 1.02–1.61). The rest of allelic combinations did not reach statistical significance in any other comparison.

## Discussion

GWAS data have confirmed *IRF5* as one of the strongest associated signals with SSc [19], [20]. In addition, it has been proposed that particular *IRF5* functional genetic elements contribute to SLE pathophysiology through their relationship with auto-antibodies and IFNα production [21], [22]. These data indicate that this gene may represent a crucial member of the genetic component underlying this type of autoimmune diseases [23].

Previous published data suggested that two different *IRF5* haplotypes influence specific SSc phenotypes. It was hypothesised that these haplotypes may explain a possible *IRF5* association with dcSSc and PF, likely by tagging an intronic 5-bp biallelic insertion-deletion polymorphism (INDEL), which would represent the real causal functional variant [12]. However, our results are not in agreement with this idea, since we did not find evidence for a specific genetic association between *IRF5* and any of the major clinical manifestations, despite the fact that two out of the three genetic variants comprising the previously described risk haplotype were covered in our analysis (rs2004640 and rs10954213 that is highly correlated with rs4728142). A similar discrepancy was also observed by Sarif *et al*. [13], who failed to replicate the *IRF5* haplotype effect on PF described by Dieudé et al. [12]. Our data are, however, consistent with recent GWAS follow-up studies that did not show a phenotype-specific association of *IRF5* with SSc, but a clear association with the overall disease [14], [24]. It should be noted that one of the SNPs included in this study, rs4728142, has been shown to correlate with longer survival and a milder pulmonary involvement in SSc patients [13]. Taking this together with our results, it could be speculated that, although the risk variants of *IRF5* do not predispose to develop PF, they may influence the severity of some clinical features like PF. In any case, it is important to note that whereas antibody profile and disease subtypes are clearly a dichotomous outcome, PF can range from mild-stable to severe-progressive involvement (and the utilised approach for definition of PF does not differentiate the different severity scales of this disease manifestation) [25].

As stated before, the tag SNPs analysed here are representative of three haplotype blocks that have been reported to affect the function of the protein in different ways, including production of an alternative spliced isoform, alteration of polyadenylation sites that leads to a shorter messenger RNA, and reduction of protein stability [11]. These three polymorphisms showed strong association signals in our study, supported by a high statistical power. Therefore, the functional alterations caused by their risk alleles may be also of high relevance in the pathogenic mechanisms that lead to SSc. However, the rs2004640 signal was dependant of that from rs4728142 in our study cohort. Hence, rs2004640 might not be an independent SSc susceptibility *locus* although it is functionally relevant, which suggests that not all functional variants in a determined risk gene may play an independent role in the associated disease. In any case, as described in SLE [11], we observed a significant additive effect amongst the three analysed SNPs because the haplotypes containing both the risk and protective alleles better explained the association between this *locus* and SSc. Hence, although no functional studies have been carried out yet to unmask the possible implication of the *IRF5* risk alleles in the SSc pathophysiology, it is likely that each one of the protein alterations described above influence the development of SSc individually, and that carrying all the three risk alleles results in a critically reduced protein function that highly increases SSc susceptibility.

In conclusion, this study clearly shows that a haplotype of three different functional genetic variants within the *IRF5* region confer susceptibility to SSc. The fact that this association is shared with SLE adds another piece of evidence to the common genetic background of both diseases, and provides a new perspective for the study of the type I IFN pathway and its implication in the development of autoimmune conditions.

## Supporting Information

File S1 **Table S1.** Overall statistical power of the study for each analysed *IRF5* genetic variant at the 5% significance level. **Table S2.** Independent analyses of *IRF5* genetic variants in Caucasian SSc patients and unaffected controls from Europe. **Table S3.** Meta-analysis of *IRF5* genetic variants comparing the main clinical phenotypes with unaffected controls. **Table S4.** Linkage disequilibrium structure of the *IRF5* region analysed in this study. **Table S5.** Pooled-analysis of different allelic combinations of the *IRF5* genomic region according to the presence/absence of specific clinical phenotypes.
(DOC)
