# Supplementary material for: The Systemic Lupus Erythematosus IRF5 Risk Haplotype Is Associated with Systemic Sclerosis
Source: PLoS One. 2013 Jan 23;8(1):e54419. doi: 10.1371/journal.pone.0054419 (PMC3553151; doi:10.1371/journal.pone.0054419)
Supplement: File S1 — Table S1. Overall statistical power of the study for each analysed IRF5 genetic variant at the 5% significance level. Table S2. Independent analyses of IRF5 genetic variants in Caucasian SSc patients and unaffected controls from Europe. Table S3. Meta-analysis of IRF5 genetic variants comparing the main clinical phenotypes with unaffected controls. Table S4. Linkage disequilibrium structure of the IRF5 region analysed in this study. Table S5. Pooled-analysis of different allelic combinations of the IRF5 genomic region according to the presence/absence of specific clinical phenotypes. (DOC) [file pone.0054419.s001.doc]

**Supplementary File S1.**

**Table S1.** Overall statistical power of the study for each analysed *IRF5* genetic variant at the 5% significance level.

| SNP | OR=1.1 | OR=1.2 | OR=1.3 |
| --- | --- | --- | --- |
| rs4728142 | 0.76 | 1.00 | 1.00 |
| rs2004640 | 0.77 | 1.00 | 1.00 |
| rs10488631 | 0.44 | 0.93 | 1.00 |

SNP, single-nucleotide polymorphism; OR, odds ratio.

**Table S2.** Independent analyses of *IRF5* genetic variants in Caucasian SSc patients and unaffected controls from Europe.

|  |  |  |  |  |  | **Genotype, N (%)** | | |  | **Allele test** | | |
| --- | --- | --- | --- | --- | --- | --- | --- | --- | --- | --- | --- | --- |
| **COHORT** | | **SNP** | **Position** | **½** | **Subgroup (N)** | **1/1** | **1/2** | **2/2** | **MAF (%)** | ***P*-value*** | ***P*_FDR_**** | **OR [CI 95%]***** |
| SPAIN | | rs4728142 | 3’UTR | A/G | Controls (n=1452) | 315 (21.69) | 737 (50.76) | 400 (27.55) | 47.07 | **0.017** | **0.026** | 1.14 [1.02-1.27] |
|  |  |  |  |  | SSc (n=1169) | 296 (25.32) | 586 (50.13) | 287 (24.55) | 50.38 |  |  |  |
|  |  | rs2004640 | Exon 1 | G/T | Controls (n=1443) | 282 (19.54) | 698 (48.37) | 463 (32.09) | 43.73 | 0.097 | 0.097 | 0.91 [0.82-1.02] |
|  |  |  |  |  | SSc (n=1169) | 192 (16.42) | 585 (50.04) | 392 (33.53) | 41.45 |  |  |  |
|  |  | rs10488631 | INDEL | C/T | Controls (n=1449) | 17 (1.17) | 226 (15.60) | 1206 (83.23) | 8.97 | **1.54E-07** | **4.63E-07** | 1.59 [1.34-1.89] |
|  |  |  |  |  | SSc (n=1175) | 23 (1.96) | 272 (23.15) | 880 (74.89) | 13.53 |  |  |  |
| GERMANY | | rs4728142 | 3’UTR | A/G | Controls (n=423) | 77 (18.20) | 207 (48.94) | 139 (32.86) | 42.67 | **0.016** | **0.016** | 1.25 [1.04-1.50] |
|  |  |  |  |  | SSc (n=544) | 126 (23.16) | 272 (50.00) | 146 (26.84) | 48.16 |  |  |  |
|  |  | rs2004640 | Exon 1 | G/T | Controls (n=409) | 101 (24.69) | 207 (50.61) | 101 (24.69) | 50.00 | **0.012** | **0.016** | 0.79 [0.66-0.95] |
|  |  |  |  |  | SSc (n=539) | 103 (19.11) | 270 (50.09) | 166 (30.80) | 44.16 |  |  |  |
|  |  | rs10488631 | INDEL | C/T | Controls (n=424) | 6 (1.42) | 70 (16.51) | 348 (82.08) | 9.67 | **6.03E-06** | **1.81E-05** | 1.89 [1.43-2.49] |
|  |  |  |  |  | SSc (n=545) | 17 (3.12) | 149 (27.34) | 379 (69.54) | 16.79 |  |  |  |
| THE NETHERLANDS | | rs4728142 | 3’UTR | A/G | Controls (n=723) | 122 (16.87) | 354 (48.96) | 247 (34.16) | 41.36 | **1.13E-04** | **1.70E-04** | 1.42 [1.19-1.69] |
|  |  |  |  |  | SSc (n=373) | 98 (26.27) | 177 (47.45) | 98 (26.27) | 50.00 |  |  |  |
|  |  | rs2004640 | Exon 1 | G/T | Controls (n=738) | 192 (26.02) | 372 (50.41) | 174 (23.58) | 51.22 | **8.62E-05** | **1.70E-04** | 0.70 [0.59-0.84] |
|  |  |  |  |  | SSc (n=366) | 71 (19.40) | 168 (45.90) | 127 (34.70) | 42.35 |  |  |  |
|  |  | rs10488631 | INDEL | C/T | Controls (n=741) | 5 (0.67) | 127 (17.14) | 609 (82.19) | 9.24 | **7.56E-04** | **7.56E-04** | 1.59 [1.21-2.09] |
|  |  |  |  |  | SSc (n=373) | 6 (1.61) | 92 (24.66) | 275 (73.73) | 13.94 |  |  |  |
| ITALY | | rs4728142 | 3’UTR | A/G | Controls (n=959) | 190 (19.81) | 458 (47.76) | 311 (32.43) | 43.69 | **3.16E-03** | **4.74E-03** | 1.24 [1.08-1.44] |
|  |  |  |  |  | SSc (n=605) | 144 (23.80) | 306 (50.58) | 155 (25.62) | 49.09 |  |  |  |
|  |  | rs2004640 | Exon 1 | G/T | Controls (n=948) | 223 (23.52) | 452 (47.68) | 273 (28.80) | 47.36 | **6.16E-03** | **6.16E-03** | 0.82 [0.71-0.94] |
|  |  |  |  |  | SSc (n=608) | 111 (18.26) | 293 (48.19) | 204 (33.55) | 42.35 |  |  |  |
|  |  | rs10488631 | INDEL | C/T | Controls (n=969) | 8 (0.83) | 121 (12.49) | 840 (86.69) | 7.07 | **4.33E-08** | **1.30E-07** | 1.95 [1.53-2.48] |
|  |  |  |  |  | SSc (n=609) | 10 (1.64) | 137 (22.50) | 462 (75.86) | 12.89 |  |  |  |
| UNITED KINGDOM | | rs4728142 | 3’UTR | A/G | Controls (n=376) | 83 (22.07) | 168 (44.68) | 125 (33.24) | 44.41 | 0.169 | 0.316 | 1.15 [0.94-1.40] |
|  |  |  |  |  | SSc (n=437) | 105 (24.03) | 208 (47.60) | 124 (28.38) | 47.83 |  |  |  |
|  |  | rs2004640 | Exon 1 | G/T | Controls (n=374) | 99 (26.47) | 166 (44.39) | 109 (29.14) | 48.66 | 0.379 | 0.379 | 0.92 [0.75-1.11] |
|  |  |  |  |  | SSc (n=440) | 107 (24.32) | 195 (44.32) | 138 (31.36) | 46.48 |  |  |  |
|  |  | rs10488631 | INDEL | C/T | Controls (n=375) | 5 (1.33) | 81 (21.60) | 289 (77.07) | 12.13 | 0.211 | 0.316 | 1.20 [0.90-1.61] |
|  |  |  |  |  | SSc (n=446) | 14 (3.14) | 99 (22.20) | 333 (74.66) | 14.24 |  |  |  |

*All P-values have been calculated for the allelic model. **Benjamini & Hochberg (1995) step-up FDR control. ***Odds ratio for the minor allele. SNP, single-nucleotide polymorphism. SSc, systemic sclerosis.

**Table S3.** Meta-analysis of *IRF5* genetic variants comparing the main clinical phenotypes with unaffected controls.

|  |  |  |  | **Genotype, N (%)** | | |  | **M-H allele test** | |
| --- | --- | --- | --- | --- | --- | --- | --- | --- | --- |
| **SNP** | **Position** | **1/2** | **Subgroup (N)** | **1/1** | **1/2** | **2/2** | **MAF (%)** | ***P*-value*** | **OR [CI 95%]**** |
| rs4728142 | 3’UTR | A/G | Controls (n=3933) | 787 (20.01) | 1924 (48.92) | 1222 (31.07) | 44.47 |  |  |
|  |  |  | lcSSc (n=2142) | 533 (24.88) | 1059 (49.44) | 550 (25.68) | 49.60 | **1.11E-07** | 1.23 [1.14-1.32] |
|  |  |  | dcSSc (n=986) | 236 (23.94) | 490 (49.70) | 260 (26.37) | 48.78 | **8.83E-04** | 1.19 [1.07-1.31] |
|  |  |  | ACA+ (n=1268) | 307 (24.21) | 658 (51.89) | 303 (23.90) | 50.16 | **3.30E-06** | 1.24 [1.13-1.36] |
|  |  |  | ATA+ (n=793) | 215 (27.11) | 378 (47.67) | 200 (25.22) | 50.95 | **1.57E-06** | 1.31 [1.17-1.46] |
|  |  |  | PF+ (n=893) | 237 (26.54) | 409 (45.80) | 247 (27.66) | 49.44 | **9.09E-05** | 1.23 [1.11-1.36] |
| rs2004640 | Exon 1 | G/T | Controls (n=3912) | 897 (22.93) | 1895 (48.44) | 1120 (28.63) | 47.15 |  |  |
|  |  |  | lcSSc (n=2143) | 406 (18.95) | 1026 (47.88) | 711 (33.18) | 42.88 | **6.92E-06** | 0.84 [0.78-0.91] |
|  |  |  | dcSSc (n=979) | 178 (18.18) | 485 (49.54) | 316 (32.28) | 42.95 | **1.05E-03** | 0.84 [0.76-0.93] |
|  |  |  | ACA+ (n=1272) | 211 (16.59) | 643 (50.55) | 418 (32.86) | 41.86 | **1.58E-05** | 0.82 [0.75-0.90] |
|  |  |  | ATA+ (n=793) | 137 (17.28) | 377 (47.54) | 279 (35.18) | 41.05 | **7.10E-06** | 0.78 [0.70-0.87] |
|  |  |  | PF+ (n=883) | 163 (18.46) | 417 (47.23) | 303 (34.31) | 42.07 | **4.37E-05** | 0.80 [0.72-0.89] |
| rs10488631 | INDEL tagger | C/T | Controls (n=3958) | 41 (1.04) | 625 (15.79) | 3292 (83.17) | 8.93 |  |  |
|  |  |  | lcSSc (n=2165) | 46 (2.12) | 494 (22.82) | 1625 (75.06) | 13.53 | **9.63E-14** | 1.56 [1.39-1.75] |
|  |  |  | dcSSc (n=983) | 24 (2.44) | 255 (25.94) | 704 (71.62) | 15.41 | **4.38E-15** | 1.79 [1.55-2.08] |
|  |  |  | ACA+ (n=1273) | 21 (1.65) | 306 (24.04) | 946 (74.31) | 13.67 | **4.06E-11** | 1.59 [1.38-1.83] |
|  |  |  | ATA+ (n=799) | 21 (2.63) | 212 (26.53) | 566 (70.84) | 15.89 | **6.05E-17** | 1.93 [1.65-2.26] |
|  |  |  | PF+ (n=886) | 19 (2.14) | 213 (24.04) | 654 (73.81) | 14.16 | **1.98E-10** | 1.65 [1.41-1.93] |

*All *P*-values have been calculated for the allelic model. **Odds ratio for the minor allele. lcSSc, limited cutaneous systemic sclerosis; dcSSc, diffuse cutaneous systemic sclerosis; ACA, anti-centromere antibody; ATA, anti-topoisomerase antibody; PF, pulmonary fibrosis. M-H, Mantel-Haenszel test under fixed effect. SNP, single-nucleotide polymorphism.

**Table S4.** Linkage disequilibrium structure of the *IRF5* region analysed in this study.

|  | **LD with rs10488631 (D' / r2)** | | | | |  | **LD with rs2004640 (D' / r^2^)** | | | | |
| --- | --- | --- | --- | --- | --- | --- | --- | --- | --- | --- | --- |
| **SNP** | **Spain** | **Germany** | **Netherlands** | **Italy** | **UK** |  | **Spain** | **Germany** | **Netherlands** | **Italy** | **UK** |
| rs4728142 | 0.55 / 0.04 | 0.74 / 0.10 | 0.82 / 0.10 | 0.71 / 0.06 | 0.81 / 0.11 |  | 0.96 / 0.65 | 0.92 / 0.65 | 0.96 / 0.67 | 0.93 / 0.61 | 0.94 / 0.68 |
| rs2004640 | 0.99 / 0.09 | 0.96 / 0.12 | 0.97 / 0.10 | 0.89 / 0.06 | 0.98 / 0.13 |  | NA | NA | NA | NA | NA |
| rs10488631 | NA | NA | NA | NA | NA |  | 0.99 / 0.09 | 0.96 / 0.12 | 0.97 / 0.10 | 0.89 / 0.06 | 0.98 / 0.13 |

LD, linkage disequilibrium.

**Table S5.** Pooled-analysis of different allelic combinations of the *IRF5* genomic region according to the presence/absence of specific clinical phenotypes.

| **Comparison (1/2)** | **Allelic combination** | **Freq. (1/2)** | ***P*-value** | **OR [95% CI]** | **BD** |
| --- | --- | --- | --- | --- | --- |
| lcSSc / dcSSc | GGT | 0.420 / 0.419 | 0.978 | 1.00 [0.90-1.12] | 0.15 |
|  | ATT | 0.351 / 0.372 | 0.145 | 0.92 [0.82-1.03] | 0.63 |
|  | ATC | 0.134 / 0.116 | 0.088 | 1.16 [0.98-1.36] | 0.69 |
|  | GTT | 0.067 / 0.065 | 0.915 | 1.02 [0.82-1.27] | 0.40 |
|  | GTC | 0.021 / 0.018 | 0.575 | 1.14 [0.77-1.70] | 0.28 |
| ACA+ / ACA- | GGT | 0.412 / 0.422 | 0.496 | 0.93 [0.75-1.15] | 0.01 |
|  | ATT | 0.378 / 0.357 | 0.162 | 1.08 [0.97-1.21] | 0.26 |
|  | ATC | 0.119 / 0.123 | 0.852 | 0.98 [0.84-1.15] | 0.65 |
|  | GTT | 0.066 / 0.067 | 0.845 | 0.97 [0.79-1.20] | 0.07 |
|  | GTC | 0.018 / 0.020 | 0.628 | 0.89 [0.61-1.31] | 0.77 |
| ATA+ / ATA- | GGT | 0.398 / 0.425 | 0.100 | 0.90 [0.80-1.02] | 0.56 |
|  | ATT | 0.365 / 0.367 | 0.840 | 0.99 [0.87-1.11] | 0.86 |
|  | ATC | 0.137 / 0.116 | 0.306 | 1.18 [0.86-1.63] | 0.01 |
|  | GTT | 0.070 / 0.065 | 0.714 | 1.05 [0.83-1.33] | 0.16 |
|  | GTC | 0.020 / 0.019 | 0.955 | 0.96 [0.62-1.49] | 0.70 |
| PF+ / PF- | GGT | 0.406 / 0.423 | 0.207 | 0.92 [0.82-1.04] | 0.92 |
|  | ATT | 0.368 / 0.371 | 0.952 | 0.99 [0.88-1.12] | 1.00 |
|  | ATC | 0.123 / 0.118 | 0.760 | 1.03 [0.86-1.24] | 0.88 |
|  | GTT | 0.074 / 0.060 | 0.041 | 1.28 [1.02-1.61] | 0.52 |
|  | GTC | 0.019 / 0.020 | 0.862 | 0.94 [0.61-1.45] | 0.37 |

Order of the SNPs: rs4728142*A/G | rs2004640*G/T | rs10488631*C/T

lcSSc, limited cutaneous systemic sclerosis; dcSSc, diffuse cutaneous systemic sclerosis; ACA, anti-centromere antibody; ATA, anti-topoisomerase antibody; PF, pulmonary fibrosis; OR, odds ratio

**Supp. Note.** *Membership of Spanish Scleroderma Group*

**Norberto Ortego-Centeno** and **Raquel Ríos**, Department of Internal Medicine, Hospital Clínico Universitario San Cecilio, Granada; **Nuria Navarrete**, Department of Internal Medicine, Hospital Virgen de las Nieves, Granada; **Rosa García Portales**, Department of Rheumatology, Hospital Virgen de la Victoria, Málaga; **Antonio Fernández-Nebro**, Department of Rheumatology, Hospital Carlos Haya, Málaga; **María F. González-Escribano**, Department of Immunology, Hospital Virgen del Rocío, Sevilla; **Julio Sánchez-Román**, **Francisco José García-Hernández** and **María Jesús Castillo**, Department of Internal Medicine, Hospital Virgen del Rocío, Sevilla; **María Ángeles Aguirre** and **Inmaculada Gómez-Gracia**, Department of Rheumatology, Hospital Reina Sofía, Córdoba; **Benjamín Fernández-Gutiérrez** and **Luis Rodríguez-Rodríguez**, Department of Rheumatology, Hospital Clínico San Carlos, Madrid; **Esther Vicente**, Department of Rheumatology, Hospital La Princesa, Madrid; **José Luis Andreu**, Department of Rheumatology, Hospital Puerta del Hierro, Madrid; **Paloma García de la Peña**, Department of Rheumatology, Hospital Madrid Norte Sanchinarro, Madrid; **Francisco Javier López-Longo** and **Lina Martínez**, Department of Rheumatology, Hospital General Universitario Gregorio Marañón, Madrid; **Vicente Fonollosa**, Department of Internal Medicine, Hospital Valle de Hebrón, Barcelona; **Gerard Espinosa**, Department of Internal Medicine, Hospital Clinic, Barcelona; **Iván Castellví**, Department of Rheumatology, Hospital de la Santa Creu i Sant Pau, Barcelona; **Carlos Tolosa**, Department of Internal Medicine, Hospital Parc Tauli, Sabadell; **Anna Pros**, Department of Rheumatology, Hospital Del Mar, Barcelona; **Mónica Rodríguez Carballeira**, Department of Internal Medicine, Hospital Universitari Mútua Terrasa, Barcelona; **Francisco Javier Narváez**, Department of Rheumatology, Hospital Universitari de Bellvitge, Barcelona; **Miguel Ángel González-Gay**, Department of Rheumatology, Hospital Universitario Marqués de Valdecilla, IFIMAV, Santander; **Bernardino Díaz**, **Luis Trapiella** and **María Gallego**, Department of Internal Medicine, Hospital Central de Asturias, Oviedo; **María del Carmen Freire** and **Inés Vaqueiro**, Unidad de Trombosis y Vasculitis, Department of Internal Medicine, Hospital Xeral-Complexo Hospitalario Universitario de Vigo, Vigo; **Federico Díaz** and **Vanesa Hernández**, Department of Rheumatology, Hospital Universitario de Canarias, Tenerife; **José Andrés Román-Ivorra**, Department of Rheumatology, Hospital Universitari i Politecnic La Fe, Valencia. **Francisco J. Blanco García** and **Natividad Oreiro**, Department of Rheumatology, INIBIC-Hospital Universitario A Coruña, La Coruña.
